# Supplementary material for: Unequal distributions of crowdsourced weather data in England and Wales
Source: Nat Commun. 2024 Jun 20;15:4828. doi: 10.1038/s41467-024-49276-z (PMC11190285; doi:10.1038/s41467-024-49276-z)
Supplement: Supplementary file 1 — Supplementary Information [file 41467_2024_49276_MOESM1_ESM.pdf]

Supplementary Information for

Unequal distributions of crowdsourced weather data in England and Wales

Oscar Brousse[1,*], Charles H. Simpson[1], Ate Poorthuis[2], and Clare Heaviside[1]

[1]University College London, Institute of Environmental Design and Engineering, London, United Kingdom, 14 Upper Woburn Pl (Central House), WC1H 0NN

[2]Katholieke Universiteit Leuven, Department of Earth and Environmental Sciences, Leuven, Belgium,Celestijnenlaan 200E, B-3001

*o.brousse@ucl.ac.uk

This file includes:

Supplementary Figures

- Supplementary Figures 1, 2, 3, 4

Supplementary Tables

- Supplementary Table 1

Supplementary Discussion

- Supplementary Figures 5, 6

Supplementary References

Supplementary Figures

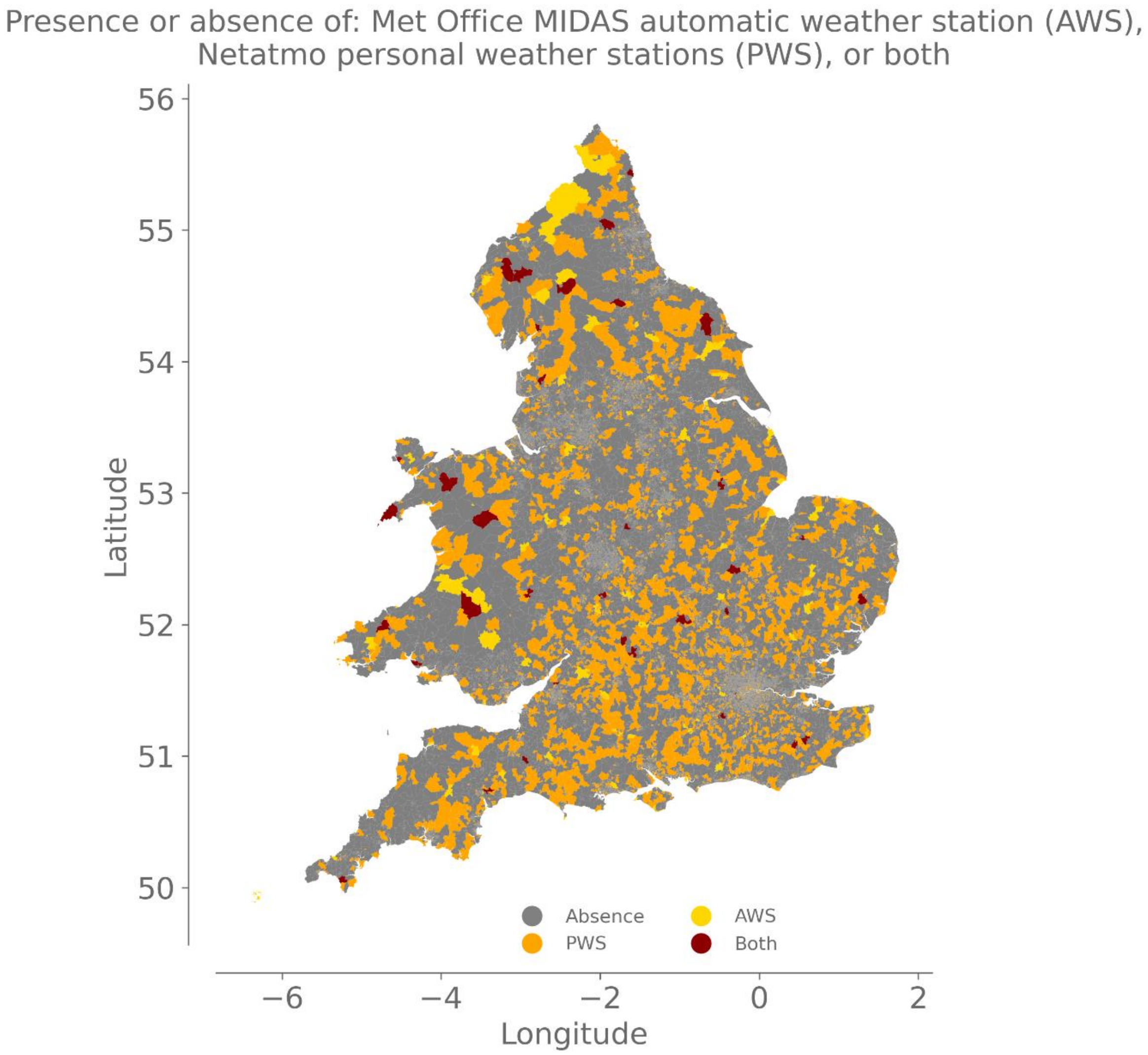

Supplementary Figure 1: Lower layer Super Output Areas where official weather stations from the United Kingdom Met Office Integrated Data Archive System (MIDAS) network (AWS) are active are in yellow, where *Netatmo* personal weather stations (PWS) are present are in orange, where both are active are in red (Both) and where none is present are shown in gray (Absence).

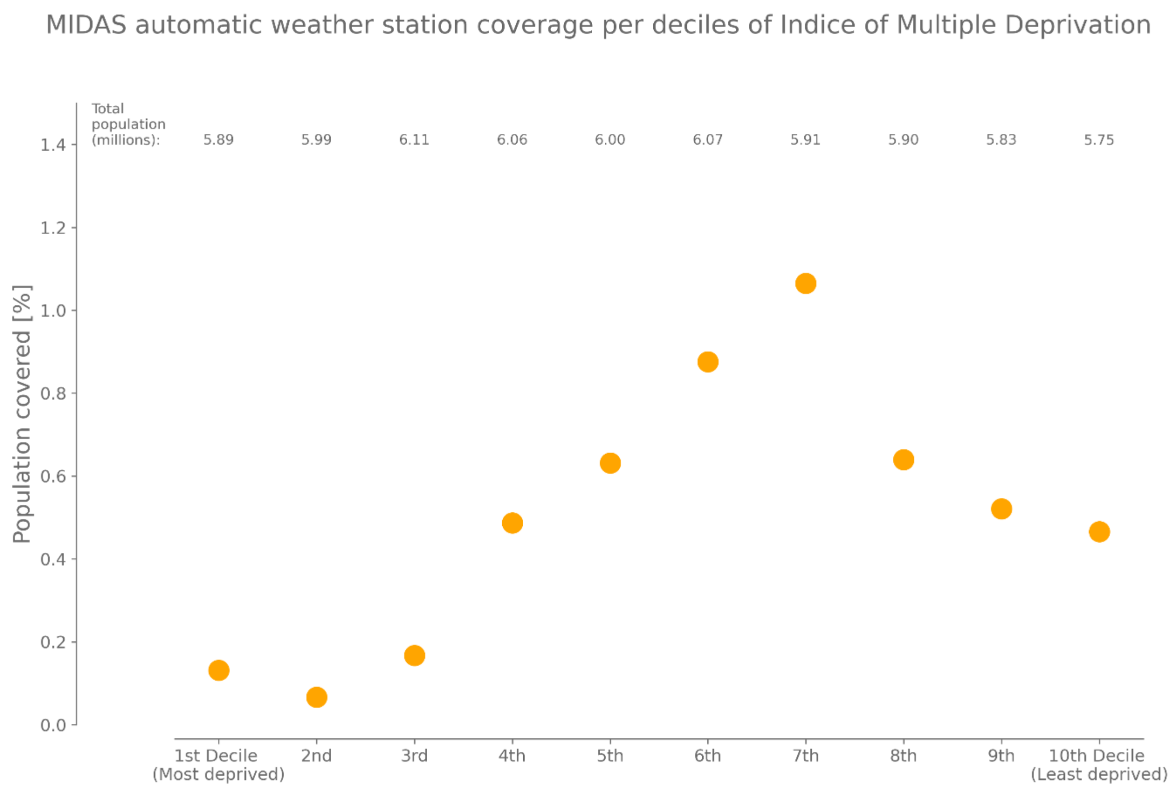

Supplementary Figure 2: Percentages of people in England and Wales covered by at least one official Met Office Integrated Data Archive System (MIDAS) automatic weather station (AWS) per Lower layer Super Output Area (LSOA), per Index of Multiple Deprivation (IMD) decile. Most deprived populations are less covered by AWS. The total number of people in each IMD decile is given at the top of the figure and shows fluctuations due to some variability in LSOA population size (~1500 inhabitants per LSOA; see Methods).

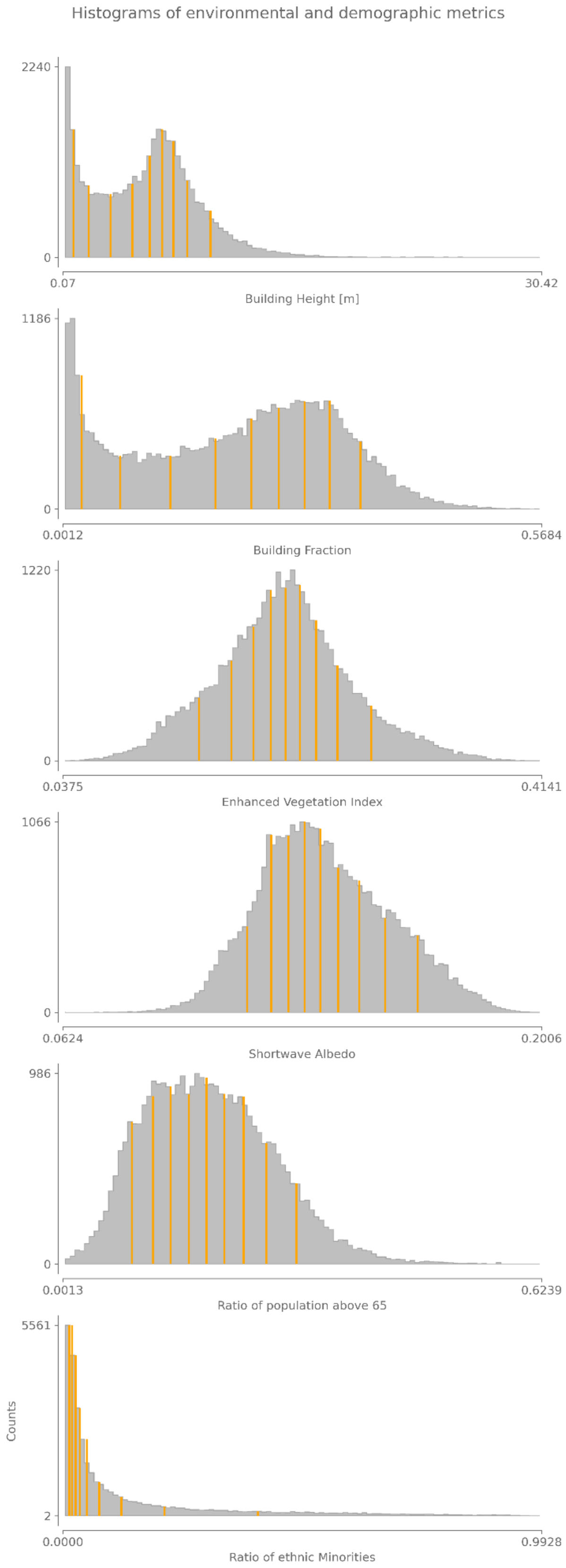

Supplementary Figure 3: Histograms using 100 bins of each demographic and environmental metrics from this study, in grey, and relative deciles' cut-off values in orange used in Figure 4.

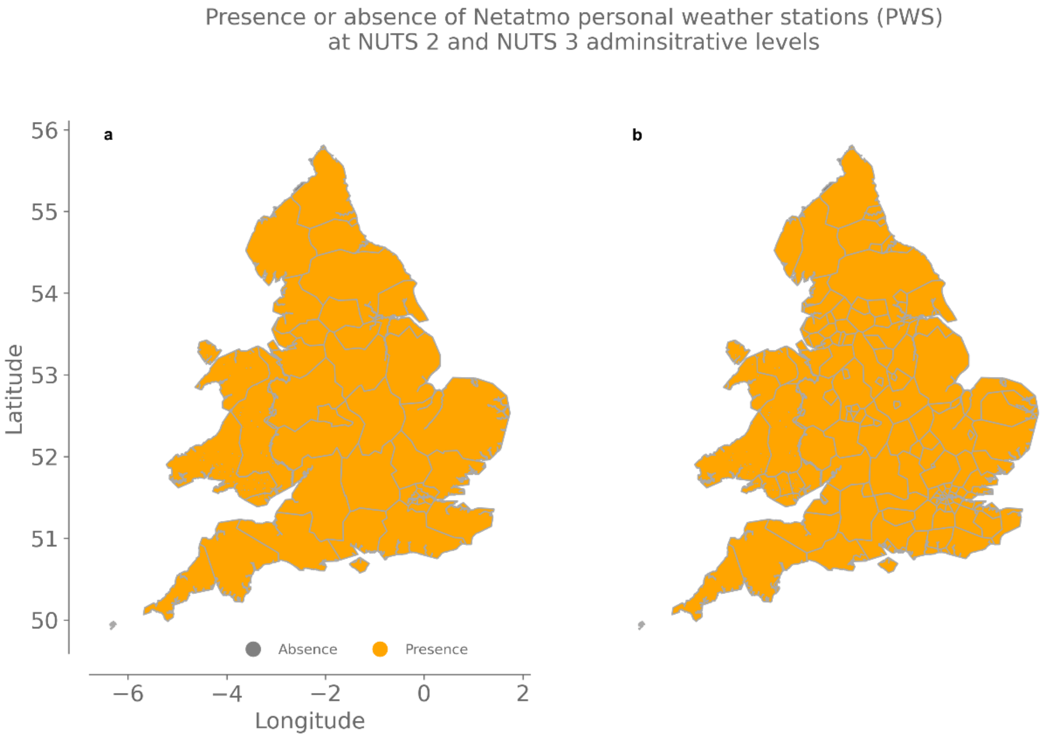

Supplementary Figure 4: Presence of at least one personal weather station (PWS) in Nomenclature of Territorial Units for Statistics (NUTS) administrative units of second (NUTS2, **a**) and third (NUTS3, **b**) levels.

Supplementary Tables

Supplementary Table 1: Average value of each demographic and environmental characteristics for all Lower layer Super Output Areas in each Index for Multiple Deprivation decile where personal weather stations (PWS) are present ($\overline{\mathbf{X}_{pws}}$) or absent ($\overline{\mathbf{X}_{abs}}$). Perkins skill scores ($\overline{\mathbf{X}_{PSS}}$; ranging from 0 to 1) between the probability density functions (PDFs) of each metric where PWS are present and absent are also given and show the degree of overlap between the two PDFs – a score over 0.7 means an important overlap.

	**1st Decile**	**2nd Decile**	**3rd Decile**	**4th Decile**	**5th Decile**	**6th Decile**	**7th Decile**	**8th Decile**	**9th Decile**	**10th Decile**
$\overline{\mathbf{BH}_{pws}}$	7.51	6.56	6.43	4.73	3.69	3.47	3.04	3.4	3.61	3.71
$\overline{\mathbf{BH}_{abs}}$	7.09	6.99	6.69	6.01	5.41	4.94	4.55	4.37	4.17	4.24
$\overline{\mathbf{BH}_{PSS}}$	0.76	0.76	0.79	0.74	0.74	0.74	0.74	0.79	0.87	0.86
$\overline{\mathbf{BF}_{pws}}$	0.27	0.23	0.23	0.17	0.14	0.13	0.12	0.14	0.15	0.15
$\overline{\mathbf{BF}_{abs}}$	0.27	0.26	0.25	0.23	0.21	0.19	0.18	0.18	0.17	0.18
$\overline{\mathbf{BF}_{PSS}}$	0.74	0.75	0.78	0.74	0.72	0.73	0.73	0.79	0.86	0.85
$\overline{\mathbf{EVI}_{pws}}$	0.18	0.19	0.2	0.22	0.24	0.24	0.25	0.24	0.24	0.25
$\overline{\mathbf{EVI}_{abs}}$	0.18	0.18	0.19	0.2	0.21	0.22	0.23	0.23	0.23	0.24
$\overline{\mathbf{EVI}_{PSS}}$	0.69	0.76	0.8	0.81	0.77	0.73	0.78	0.83	0.86	0.86
$\overline{\mathbf{Alb}_{pws}}$	0.13	0.13	0.13	0.14	0.15	0.15	0.15	0.15	0.15	0.15
$\overline{\mathbf{Alb}_{abs}}$	0.13	0.13	0.13	0.14	0.14	0.14	0.14	0.14	0.14	0.14
$\overline{\mathbf{Alb}_{PSS}}$	0.77	0.79	0.81	0.76	0.76	0.75	0.76	0.8	0.86	0.89
$\overline{\mathbf{P65}_{pws}}$	0.14	0.16	0.17	0.2	0.22	0.22	0.23	0.23	0.23	0.24
$\overline{\mathbf{P65}_{abs}}$	0.13	0.14	0.16	0.18	0.2	0.21	0.22	0.23	0.23	0.24
$\overline{\mathbf{P65}_{PSS}}$	0.72	0.78	0.79	0.82	0.8	0.84	0.84	0.9	0.88	0.89
$\overline{\mathbf{Eth}_{pws}}$	0.16	0.18	0.18	0.13	0.08	0.08	0.06	0.07	0.06	0.06
$\overline{\mathbf{Eth}_{abs}}$	0.2	0.22	0.21	0.17	0.14	0.12	0.1	0.08	0.07	0.07
$\overline{\mathbf{Eth}_{PSS}}$	0.74	0.79	0.77	0.82	0.82	0.84	0.87	0.91	0.91	0.9

Supplementary Discussion

Modal Local Climate Zone and loss of information

Characterising each Lower layer Super Output Area (LSOA) by a single Local Climate Zone necessarily comes with a simplification of the complexity of the environment that composes each LSOA; something natural to any classification exercise. In this study, we chose to classify each LSOA in the form of Local Climate Zone by choosing the modal LCZ as the determining entity of the LSOA's *urban climatic* background. The modal LCZ is the LCZ that composes most of the LSOA area (Supplementary Figure 5). This could therefore come with an over simplification of the LSOA environment in cases where, for example, LSOAs are composed of two major LCZs that are only separated by a few percentage points in their proportion of the covered area (e.g., 48 % vs 52 %). Below, we show how choosing the modal LCZ as a class for the entire LSOA is expected to have a low impact on the conclusions presented in this study.

In England and Wales, as higher population densities are found around cities, urban LSOAs tend to be smaller than their natural counterparts. This minimises the chances of having multiple disparate LCZs within urban LSOAs as can be seen in Supplementary Figure 5 (e.g., panels **a** to **f** and **h**). As population density lowers in rural areas (Supplementary Figure 5 panels **g** and **f** to **o**) LSOAs cover larger areas and are therefore more prone to a plurality of LCZs that compose their territory; despite the hegemony of *LCZ D: Low Plants* (Supplementary Figure 5**l**) in rural England and Wales.

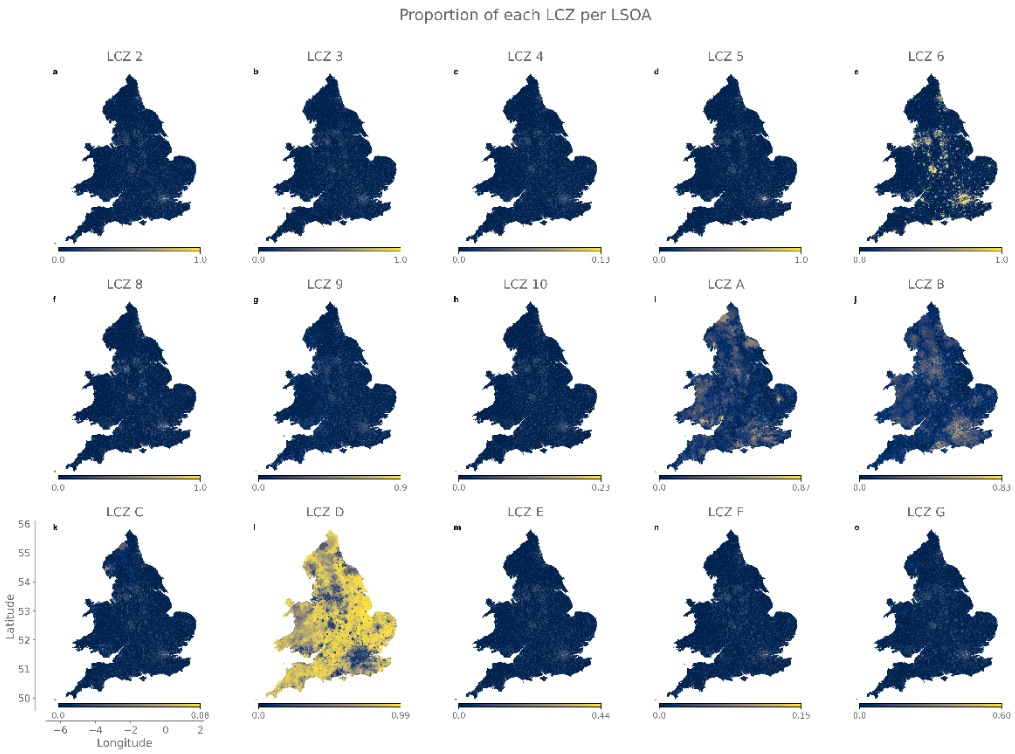

Supplementary Figure 5: Proportions of each Local Climate Zone (LCZ; **a** to **o**) composing the land-use land-cover of England and Wales in each Lower layer Super Output Area (LSOA). LCZs are extracted from the European LCZ map from Demuzere et al. (2019). For the LCZ codification, please refer to the main manuscript or Supplementary Figure 6, below.

By plotting the cumulative density function of proportions of other LCZ in each modal LCZ at the LSOA level (Supplementary Figure 6), we find that other LCZs do not compose more than a 1/3 of the LSOA area in more than 80 % of the cases (except for LCZ 6 in LSOAs classified as LCZ 3, LCZ 8 and LCZ B (Supp Figure 6**b**, **e** and **h**), remembering that LCZ 6 (n= 22529) composes most of the LSOAs in England and Wales with LCZ D (n=8027). This shows that in a vast majority the modal LCZ is unanimously the one that composes the larger part of each LSOA and can therefore be expected to have the greatest influence on the local climate and characterise it. Withal, other prominent LCZs in each LSOA are of similar types than the modal LCZ in which they are embedded. For example, urban *Compact Low-Rises* (LCZ 3) are generally found in greater proportions in LSOAs classified as *Compact Mid-Rises* (LCZ 2; Supplementary Figure 6**a**). This means that independent of the presence of other LCZs in each LSOA, a generic typology of local climate can be expected, such as: compact urban, open urban, sparsely built and natural (forested or afforested).

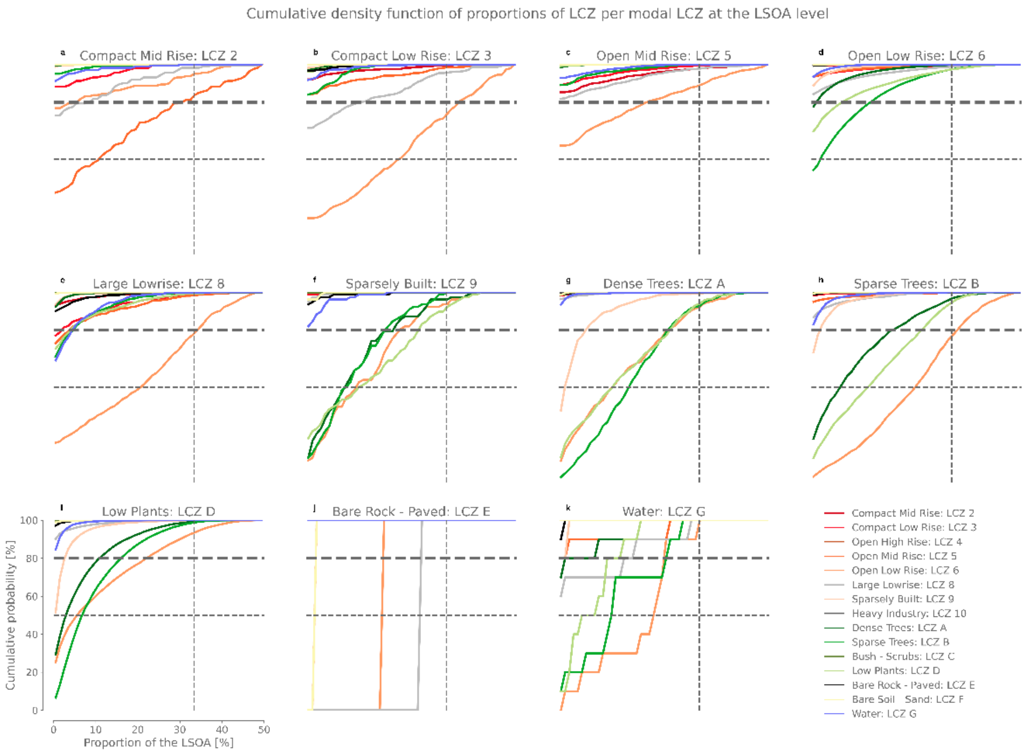

Supplementary Figure 6: Cumulative distributions of proportions of other Local Climate Zones (LCZs) composing Lower layer Super Output Areas (LSOAs) classified as LCZ_X using a modal classification (**a** to **k**). Distributions are from 50 bin histograms spanning proportions from 0 % to 50 %; above 50 %, the LCZ would necessarily become modal. The horizontal light dashed line indicates that half of LCZ_X are lower or greater than a proportion *p*. The horizontal bold dashed line indicates the same but with 80 % of the proportions being lower than *p*. The vertical dashed line shows a proportion *p* equal to 1/3 of the LSOA area.

Our results are in line with previous studies from Bechtel et al. (2017) which showed that there is a greater chance of having similar LCZ clustered together; this supports the development of the weighted accuracy for evaluating LCZ maps' accuracy. By considering "how wrong" our modal classification is, to cite Bechtel et al. (2020), we argue that our modal classification certainly misses part of the LSOAs' climatic environment complexity. At the same time; the

expected climate from the modal LCZ is not thought to be entirely dissimilar to one that would consider the variety of LCZs that compose each LSOA. For the purpose of our study, which is simply to characterise the typology of local climates that are currently being sensed at the national scale, we believe that the modal LCZ is therefore sufficient. Other studies that would try to predict air temperature at the LSOA level or to investigate the causal explanations of the personal weather station density are encouraged to use more discrete variables like the proportion of LCZ.

Supplementary References

1. Bechtel, B. *et al*. Quality of crowdsourced data on urban morphology—the human influence experiment (HUMINEX). *Urban Science*, **1**, 15 (2017).

2. Bechtel, B., Demuzere, M. & Stewart, I. D. A weighted accuracy measure for land cover mapping: comment on Johnson et al. local climate zone (LCZ) map accuracy assessments should account for land cover physical characteristics that affect the local thermal environment. *Remote Sensing*, **12**, 1769 (2020).

3. Demuzere, M., Bechtel, B., Middel, A. & Mills, G. Mapping Europe into local climate zones. *PloS one*, **14**(4), e0214474 (2019).
