# Supplementary material for: Unequal distributions of crowdsourced weather data in England and Wales
Source: Nat Commun. 2024 Jun 20;15:4828. doi: 10.1038/s41467-024-49276-z (PMC11190285; doi:10.1038/s41467-024-49276-z)
Supplement: Supplementary file 3 — Reporting Summary [file 41467_2024_49276_MOESM3_ESM.pdf]

nature portfolio

Corresponding author(s): Oscar Brousse

Last updated by author(s): Apr 10, 2024

Reporting Summary

Nature Portfolio wishes to improve the reproducibility of the work that we publish. This form provides structure for consistency and transparency in reporting. For further information on Nature Portfolio policies, see our Editorial Policies and the Editorial Policy Checklist.

Statistics

For all statistical analyses, confirm that the following items are present in the figure legend, table legend, main text, or Methods section.

n/a	Confirmed	
☐	☒	The exact sample size (*n*) for each experimental group/condition, given as a discrete number and unit of measurement
☐	☒	A statement on whether measurements were taken from distinct samples or whether the same sample was measured repeatedly
☒	☐	The statistical test(s) used AND whether they are one- or two-sided<br>*Only common tests should be described solely by name; describe more complex techniques in the Methods section.*
☐	☒	A description of all covariates tested
☐	☒	A description of any assumptions or corrections, such as tests of normality and adjustment for multiple comparisons
☒	☐	A full description of the statistical parameters including central tendency (e.g. means) or other basic estimates (e.g. regression coefficient) AND variation (e.g. standard deviation) or associated estimates of uncertainty (e.g. confidence intervals)
☒	☐	For null hypothesis testing, the test statistic (e.g. *F*, *t*, *r*) with confidence intervals, effect sizes, degrees of freedom and *P* value noted<br>*Give P values as exact values whenever suitable.*
☒	☐	For Bayesian analysis, information on the choice of priors and Markov chain Monte Carlo settings
☒	☐	For hierarchical and complex designs, identification of the appropriate level for tests and full reporting of outcomes
☒	☐	Estimates of effect sizes (e.g. Cohen's *d*, Pearson's *r*), indicating how they were calculated

Our web collection on statistics for biologists contains articles on many of the points above.

Software and code

Policy information about availability of computer code

Data collection	The European continental LCZ map was directly downloaded from the World Urban Database and Access Portal Tool website at: https://www.wudapt.org/lcz-maps/ . MODIS Terra Daily Enhanced Vegetation Index (EVI) was obtained from Google's Earth Engine: https://developers.google.com/earth-engine/datasets/catalog/MODIS_MOD09GA_006_EVI . MCD43A3.061 shortwave MODIS Albedo Daily 500m was obtained from: https://developers.google.com/earth-engine/datasets/catalog/MODIS_061_MCD43A3 . Global Human Settlement Layers built-up surfaces (GHS-BUILT-S) and built-up heights (GHS-BUILT-H) was obtained from the European Commission website: https://human-settlement.emergency.copernicus.eu/download.php . The ethnic groups were obtained from the Office for National Statistics 2011 census at: https://www.nomisweb.co.uk/census/2011/qs201ew . The population (age and total population per Lower Layer Super Output area) was obtained from the Office for National Statistics 2011 census at : https://www.nomisweb.co.uk/census/2011/ks102ew . The 2019 Index of Multiple Deprivation (IMD) for England and Wales was obtained from the Consumer Data Research Centre (CDRC) datasets : https://data.cdrc.ac.uk/dataset/index-multiple-deprivation-imd . The Met Office Integrated Data Archive System (MIDAS) metadata was obtained from the Centre for Environmental Data Analysis (CEDA) Archive : https://catalogue.ceda.ac.uk/uuid/220a65615218d5c9cc9e4785a3234bd0 . We used the secured FTP protocol to download it using WinSCP -- this requires an accredited account from CEDA; more info is available here: https://help.ceda.ac.uk/article/280-ftp . The Netatmo metadata was obtained using the "patatmo" Python API which requires the users to be Netatmo App developers (see https://dev.netatmo.com/). All necessary information and links for installation are available at: https://nobodyinperson.gitlab.io/python3-patatmo/ .
Data analysis	We used Python v3.9.15 on Visual Studio Code to perform our analysis.

For manuscripts utilizing custom algorithms or software that are central to the research but not yet described in published literature, software must be made available to editors and reviewers. We strongly encourage code deposition in a community repository (e.g. GitHub). See the Nature Portfolio guidelines for submitting code & software for further information.

Data

Policy information about availability of data

All manuscripts must include a data availability statement. This statement should provide the following information, where applicable:

- Accession codes, unique identifiers, or web links for publicly available datasets
- A description of any restrictions on data availability
- For clinical datasets or third party data, please ensure that the statement adheres to our policy

All data used in this study have been uploaded on a public GitHub repository: https://github.com/oscarbrousse/NatComms_PWS_2024.

We collected the data as follows:
The European continental LCZ map was directly downloaded from the World Urban Database and Access Portal Tool website at: https://www.wudapt.org/lcz-maps/ . MODIS Terra Daily Enhanced Vegetation Index (EVI) was obtained from Google's Earth Engine: https://developers.google.com/earth-engine/datasets/catalog/MODIS_MOD09GA_006_EVI . MCD43A3.061 shortwave MODIS Albedo Daily 500m was obtained from: https://developers.google.com/earth-engine/datasets/catalog/MODIS_061_MCD43A3 . Global Human Settlement Layers built-up surfaces (GHS-BUILT-S) and built-up heights (GHS-BUILT-H) was obtained from the European Commission website: https://human-settlement.emergency.copernicus.eu/download.php . The ethnic groups were obtained from the Office for National Statistics 2011 census at: https://www.nomisweb.co.uk/census/2011/qs201ew . The population (age and total population per Lower Layer Super Output area) was obtained from the Office for National Statistics 2011 census at : https://www.nomisweb.co.uk/census/2011/ks102ew . The 2019 Index of Multiple Deprivation (IMD) for England and Wales was obtained from the Consumer Data Research Centre (CDRC) datasets : https://data.cdrc.ac.uk/dataset/index-multiple-deprivation-imd . The Met Office Integrated Data Archive System (MIDAS) metadata was obtained from the Centre for Environmental Data Analysis (CEDA) Archive : https://catalogue.ceda.ac.uk/uuid/220a65615218d5c9cc9e4785a3234bd0 . We used the secured FTP protocol to download it using WinSCP -- this requires an accredited account from CEDA; more info is available here: https://help.ceda.ac.uk/article/280-ftp . The Netatmo metadata was obtained using the "patatmo" Python API which requires the users to be Netatmo App developers (see https://dev.netatmo.com/). All necessary information and links for installation are available at: https://nobodyinperson.gitlab.io/python3-patatmo/ .

MIDAS and \textit{Netatmo} stations metadata can be obtained on the dedicated websites and through their relative APIs, as explained above. The raw Netatmo and MIDAS metadata are protected and cannot be openly shared. The codes with all publicly sharable data has been released under the Zenodo repository with DOI: 10.5281/zenodo.10950425 ; the latter is linked to the public GitHub repository.

Metadata files used in this study by the authors can be obtained upon reasonable request to Dr. Oscar Brousse (o.brousse@ucl.ac.uk).

Research involving human participants, their data, or biological material

Policy information about studies with human participants or human data. See also policy information about sex, gender (identity/presentation), and sexual orientation and race, ethnicity and racism.

Reporting on sex and gender	N/A
Reporting on race, ethnicity, or other socially relevant groupings	N/A
Population characteristics	N/A
Recruitment	N/A
Ethics oversight	N/A

Note that full information on the approval of the study protocol must also be provided in the manuscript.

Field-specific reporting

Please select the one below that is the best fit for your research. If you are not sure, read the appropriate sections before making your selection.

☐ Life sciences ☐ Behavioural & social sciences ☒ Ecological, evolutionary & environmental sciences

For a reference copy of the document with all sections, see nature.com/documents/nr-reporting-summary-flat.pdf

Ecological, evolutionary & environmental sciences study design

All studies must disclose on these points even when the disclosure is negative.

Study description	We describe the underlying environment of where personal weather stations from the Netatmo company are located in England and Wales using a set of variables aggregated at the Lower layer Super Output Area (LSOA) that are relevant to heat vulnerability: land-use land-cover, environmental, socio-economic and demographic. Our analysis is based on single variable comparisons that are hierarchically ordered using deciles.

Research sample	We constrain our analysis to England and Wales to have consistency between our 2019 Index of Multiple Deprivation dataset and chose to aggregate all our data at the Lower layer Super-Output Area level to benefit from the details provided at the smallest level of administrative unit where our data was accessible in England and Wales. This means that our data is representative of a total population of n=59,513,320 inhabitants in 2011 (following the last census available at the time of the study) and where n=5011 personal weather stations were accessible for the year 2022.
Sampling strategy	There were no sample size calculation involved in our study. We believe that the total amount of LSOAs existing in England and Wales (n=34,632) and our sample of Netatmo personal weather stations (n=5011) are sufficient to draw the conclusions of our study.
Data collection	The data was downloaded from the relevant websites described above and in the "Data Availability" statement. The data was collected by the lead author, Dr. Oscar Brousse, and by the second author, Dr. Charles Simpson. The data was stored on their personal professional laptops. It was also shared between co-authors (including Prof. Ate Poorthuis and Dr. Clare Heaviside) via a private GitHub repository. The protected metadata for Netatmo personal weather stations and MIDAS official weather stations is stored by Dr. Oscar Brousse and can be shared upon reasonable request (e.g., reproducibility)
Timing and spatial scale	The data was punctually acquired from the archives provided in the "Data Availability" statement. The first download was performed on the 22nd of October 2022 and data availability was last checked on the 4th of March 2024. To the authors' knowledge, no major data alteration (e.g., quality check, periodical releases, etc.) were performed since the data was first accessed; sampling date has therefore minimal chances to have an impact on the outcome of the study. We always used the most recent dataset available at the time of our study. The census tract data is valid for 2011, the IMD is valid for 2019, the Local Climate Zone map for 2019, the median MODIS EVI and shortwave albedo are representative of the years 2018 to 2022 (5-year average; representative for the other environmental conditions), the GHS building height is representative of 2018 and the built-up surface of 2020. The MIDAS official weather station network was active during the summer 2022. The Netatmo network metadata was only gathered for the Summer 2022 (1st of June to 1st of September 2022) as this was the a record-breaking year in daily maximum temperature in England and Wales. That data collection was performed on the 22nd of October 2022. We wanted to give an overview of the crowdsourced temperature sensor coverage at that point.<br><br>Concerning the spatial resolution, all our demographic and socio-economic data are at the LSOA level; polygons covering ~1500 inhabitants in England and Wales. We aggregate our environmental data to these polygons. The initial spatial resolution was the highest available: 500 m for MODIS products, 100 m for LCZ, and 100 m for GHSL. The Netatmo and MIDAS metadata are points localized by latitude and longitude and transformed into "presence/absence" at the LSOA level.<br><br>All the relevant information on the rationale is given in the Methods of the manuscript.
Data exclusions	We excluded the Netatmo and the MIDAS weather stations that were not active during the summer 2022 from the analysis. MIDAS weather stations located offshore were also excluded as our study focuses on England and Wales land. No data was excluded for the other variables.
Reproducibility	Our study is entirely reproducible with the same data. Collection of Netatmo personal weather stations' metadata can nonetheless change depending on sensors' connectivity to the server. This should have minor impact on the outcome of the study.<br><br>Co-authors shared the codes and data together to make sure that the outcomes were reproducible between their machines. The official code used for the study is flagged at the zenodo directory with DOI: 10.5281/zenodo.10950425. Upon request to the corresponding author Dr. Oscar Brousse, data can be shared to validate the reproducibility.
Randomization	The data has been aggregated at Lower Layer Super Output areas (LSOAs) and grouped by deciles for the analysis. We simply perform a one to one comparison of our variables. Randomization is thus not required.
Blinding	Unconscious and unreported human influence, or biases, on our study are absent or at least minimal. We provide a straight forward methodology, extensively detailed in the dedicated section, to directly use existing data and draw conclusions out of them. The codes are publicly available for reproducibility. Hence blinding is not necessary.

Did the study involve field work? ☐ Yes ☒ No

Reporting for specific materials, systems and methods

We require information from authors about some types of materials, experimental systems and methods used in many studies. Here, indicate whether each material, system or method listed is relevant to your study. If you are not sure if a list item applies to your research, read the appropriate section before selecting a response.

Materials & experimental systems

n/a	Involved in the study
☒	☐ Antibodies
☒	☐ Eukaryotic cell lines
☒	☐ Palaeontology and archaeology
☒	☐ Animals and other organisms
☒	☐ Clinical data
☒	☐ Dual use research of concern
☒	☐ Plants

Methods

n/a	Involved in the study
☒	☐ ChIP-seq
☒	☐ Flow cytometry
☒	☐ MRI-based neuroimaging

Plants

Seed stocks	N/A
Novel plant genotypes	N/A
Authentication	N/A
